# Supplementary material for: Noncanonical mechanism of voltage sensor coupling to pore revealed by tandem dimers of Shaker
Source: Nat Commun. 2019 Aug 8;10:3584. doi: 10.1038/s41467-019-11545-7 (PMC6687735; doi:10.1038/s41467-019-11545-7)
Supplement: Supplementary file 1 — Supplementary Information [file 41467_2019_11545_MOESM1_ESM.docx]

**Non-canonical mechanism of voltage sensor coupling to pore revealed by tandem dimers**

Joao L Carvalho-de-Souza^1#^ and Francisco Bezanilla^1,2,*^

^1^Department of Biochemistry and Molecular Biology, The University of Chicago, Chicago, IL, USA, 60637.

^2^Centro Interdisciplinario de Neurociencia de Valparaíso, Facultad de Ciencias, Universidad de Valparaíso, Valparaíso, Chile

^#^current address: Department of Anesthesiology, University of Arizona, Tucson, AZ.

* Corresponding Author; fbezanilla@uchicago.edu

**SUPPLEMENTARY INFORMATION**

**SUPPLEMENTARY TABLES**

**Table 1.** Voltage dependent parameters of Shaker and Shaker dimers containing only wild-type VSDs.

|  | CONSTRUCTS | V_1/2_ | V_0_ | V_1_ | V_0*_ | V_1*_ | V_inact_ |
| --- | --- | --- | --- | --- | --- | --- | --- |
| Shaker | VSD^wt^PD^wt^ | -22.6 ± 0.34 |  |  |  |  | -30.42 ± 0.12 |
| Non-conductive Shaker | VSD^wt^PD^W434F^ |  | -53.5 ± 1.91 | -47.0 ± 1.17 |  |  |  |
| Wild-type VSD Shaker dimers | VSD^wt^PD^wt^−VSD^wt^PD^wt^ | -20.7 ± 0.79 |  |  |  |  | -26.8 ± 0.14 |
|  | VSD^wt^PD^wt^−VSD^wt^PD^W434F^ | -22.0 ± 0.93 |  |  |  |  | -37.52 ± 0.31 |
|  | VSD^wt^PD^W434F^−VSD^wt^PD^wt^ | -21.1 ± 0.74 |  |  |  |  | -48.02 ± 0.27 |

Constructs column: VSD^x^PD^y^ (regular Shaker – x: VSD mutation; y: PD mutation); VSD^x1^PD^y1^−VSD^x2^PD^y2^ (Shaker dimers – x1: VSD mutation in protomer 1; y1: PD mutation in protomer 1; x1: VSD mutation in protomer 2; y1: PD mutation in protomer 2)

**Table 2.** Voltage dependent parameters of Shaker and Shaker dimers containing mutant VSDs.

|  | CONSTRUCT | V_1/2_ | V_0_ | V_1_ | V_0*_ | V_1*_ | V_inact_ |
| --- | --- | --- | --- | --- | --- | --- | --- |
| Mutant VSD Shaker | VSD^358E^PD^wt^ | -52.9 ± 1.11 |  |  |  |  |  |
|  | VSD^358R^PD^wt^ | -49.6 ± 0.80 |  |  |  |  |  |
|  | VSD^358W^PD^wt^ | -0.9 ± 1.32 |  |  |  |  |  |
|  | VSD^361E^PD^wt^ | -53.1 ± 1.64 |  |  |  |  |  |
|  | VSD^361R^PD^wt^ | -90.1 ± 0.48 |  |  |  |  |  |
|  | VSD^361A^PD^wt^ | +41.2 ± 0.20 |  |  |  |  |  |
|  | VSD^ILT^PD^wt^ | +89.5 ± 0.26 |  |  |  |  |  |
| Mutant VSD  non-conductive Shaker | VSD^358E^PD^W434F^ |  | -137.1 ± 2.06 | -92.1±2.19 |  |  |  |
|  | VSD^358R^PD^W434F^ |  | -125.3 ± 1.17 | -70.6±1.31 |  |  |  |
|  | VSD^358W^PD^W434F^ |  | -79.7 ± 0.71 | -35.3±0.24 |  |  |  |
|  | VSD^361E^PD^W434F^ |  | -92.5 ± 8.88 | -78.2±6.69 |  |  |  |
|  | VSD^361R^PD^W434F^ |  | -103.2 ± 1.15 | -57.4±0.97 |  |  |  |
|  | VSD^361A^PD^W434F^ |  | -90.6 ± 1.08 | +26.0±3.41 |  |  |  |
|  | VSD^ILT^PD^W434F^ |  | -79.6 ± 0.21 | +34.8±1.74 |  |  |  |
| Mutant VSD  wt-PD Shaker dimers | VSD^wt^PD^wt^−VSD^358E^PD^wt^ | -45.0 ± 0.41 |  |  |  |  |  |
|  | VSD^wt^PD^wt^−VSD^358R^PD^wt^ | -39.9 ± 0.73 |  |  |  |  |  |
|  | VSD^wt^PD^wt^−VSD^358W^PD^wt^ | -16.6 ± 1.00 |  |  |  |  |  |
|  | VSD^wt^PD^wt^−VSD^361E^PD^wt^ | -43.1 ± 0.39 |  |  |  |  |  |
|  | VSD^wt^PD^wt^−VSD^361R^PD^wt^ | -39.7 ± 0.87 |  |  |  |  |  |
|  | VSD^wt^PD^wt^−VSD^361A^PD^wt^ | +0.1 ± 0.87 |  |  |  |  |  |
|  | VSD^wt^PD^wt^−VSD^ILT^PD^wt^ | 32.2 ± 0.85 |  |  |  |  |  |
| Mutant VSD  W434F-Shaker dimers  Near configuration | VSD^wt^PD^W434F^−VSD^358E^PD^wt^ | -43.6 ± 0.97 |  |  |  |  | -83.4 ± 0.44 |
|  | VSD^wt^PD^W434F^−VSD^358R^PD^wt^ | -27.3 ± 1.15 |  |  |  |  | -63.1 ± 0.40 |
|  | VSD^wt^PD^W434F^−VSD^358W^PD^wt^ | -2.7 ± 1.09 |  |  |  |  | -30.0 ± 0.34 |
|  | VSD^wt^PD^W434F^−VSD^361E^PD^wt^ | -41.5 ± 1.24 |  |  |  |  | -71.1 ± 0.14 |
|  | VSD^wt^PD^W434F^−VSD^361R^PD^wt^ | -50.1 ± 1.06 |  |  |  |  | -79.5 ± 0.23 |
|  | VSD^wt^PD^W434F^−VSD^361A^PD^wt^ | +13.0 ± 0.84 |  |  |  |  | -47.1 ± 7.71§†  -9.3 ± 3.48‡ |
|  | VSD^wt^PD^W434F^−VSD^ILT^PD^wt^ | +54.7 ± 1.36 |  |  |  |  | -64.6 ± 0.44†  +34.1 ± 0.68‡ |
|  | VSD^ILT^PD^wt^−VSD^wt^PD^W434F^ | +62.7 ± 1.82 |  |  |  |  | -57.37 ± 2.02†  +42.74 ± 0.70‡ |
| Mutant VSD  W434F-Shaker dimers  Far configuration | VSD^wt^PD^wt^−VSD^358E^PD^W434F^ | -36.8 ± 0.89 |  |  |  |  | -47.77 ± 0.23 |
|  | VSD^wt^PD^wt^−VSD^358R^PD^W434F^ | -34.4 ± 1.09 |  |  |  |  | -48.44 ± 0.44 |
|  | VSD^wt^PD^wt^−VSD^358W^PD^W434F^ | -20.0 ± 0.58 |  |  |  |  | -45.74 ± 0.28 |
|  | VSD^wt^PD^wt^−VSD^361E^PD^W434F^ | -30.1 ± 1.10 |  |  |  |  | -48.82 ± 0.19 |
|  | VSD^wt^PD^wt^−VSD^361R^PD^W434F^ | -28.5 ± 0.93 |  |  |  |  | -53.9 ± 0.81 |
|  | VSD^wt^PD^wt^−VSD^361A^PD^W434F^ | -17.3 ± 0.79 |  |  |  |  | -39.95 ± 0.37 |
|  | VSD^wt^PD^wt^−VSD^ILT^PD^W434F^ | -16.0 ± 1.36 |  |  |  |  | -27.56 ± 0.53 |
|  | VSD^ILT^PD^W434F^−VSD^wt^PD^wt^ | +18.9 ± 1.42 |  |  |  |  | -39.97 ± 0.70 |
| Mutant VSD  non-conductive Shaker dimers | VSD^wt^PD^W434F^−VSD^wt^PD^W434F^ |  |  |  | -52.3 ± 0.84 | -45.5 ± 0.60 |  |
|  | VSD^wt^PD^W434F^−VSD^358E^PD^W434F^ |  |  |  | -99.2 ± 1.62 | -48.9 ± 0.98 |  |
|  | VSD^wt^PD^W434F^−VSD^358R^PD^W434F^ |  |  |  | -91.6 ± 1.04 | -51.1 ± 0.52 |  |
|  | VSD^wt^PD^W434F^−VSD^358W^PD^W434F^ |  |  |  | -65.7 ± 0.85 | -35.6 ± 0.47 |  |
|  | VSD^wt^PD^W434F^−VSD^361E^PD^W434F^ |  |  |  | -100.9 ± 0.56 | -52.8 ± 0.43 |  |
|  | VSD^wt^PD^W434F^−VSD^361R^PD^W434F^ |  |  |  | -66.8 ± 2.94 | -54.4 ± 1.35 |  |
|  | VSD^wt^PD^W434F^−VSD^361A^PD^W434F^ |  |  |  | -81.1 ± 0.54 | -35.5 ± 0.46 |  |
|  | VSD^wt^PD^W434F^−VSD^ILT^PD^W434F^ |  |  |  | -82.1 ± 2.30 | -51.6 ± 1.94 |  |

Constructs column: VSD^x^PD^y^ (regular Shaker – x: VSD mutation; y: PD mutation); VSD^x1^PD^y1^−VSD^x2^PD^y2^ (Shaker dimers – x1: VSD mutation in protomer 1; y1: PD mutation in protomer 1; x1: VSD mutation in protomer 2; y1: PD mutation in protomer 2)

V_1/2_: Voltage midpoint in G-V curves

V_0_: Voltage midpoint of the first component of Q-V curves from Shaker

V_1_: Voltage midpoint of the second component of Q-V curves from Shaker

V_0*_: Voltage midpoint of the first component of Q-V curves from non-conductive Shaker dimers

V_1*_: Voltage midpoint of the second component of Q-V curves from non-conductive Shaker dimers

V_inact_: Voltage midpoint of the inactivation curves

† First component of split inactivation curve

‡ Second component of split inactivation curve

**Table 3.** Voltage dependent parameters of Shaker and Shaker S412V.

|  | V_0_ | V_1_ | V_1/2_ |
| --- | --- | --- | --- |
| Shaker (wt) | -54.55 | -48.34 | -22.80 |
| Shaker S412V | -39.50 | -17.55 | +15.40 |

V_0_ and V_1_: Average value in mV of the voltage dependent parameters from Q-V curves fitted by a three-state model.

V_1/2_: Average value in mV of the voltage dependent parameters from G-V curves fitted by a two-state model.

**SUPPLEMENTARY FIGURES**


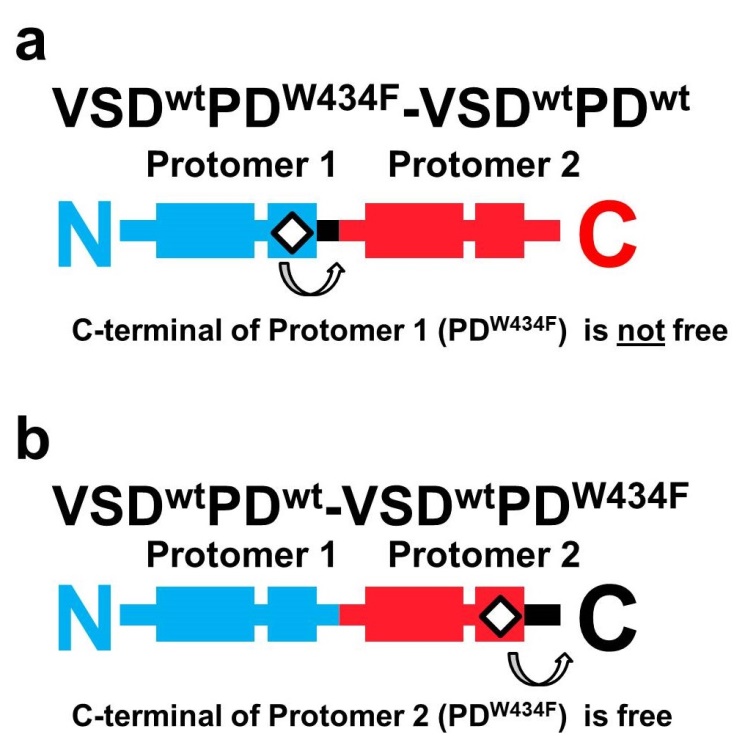


**Supplementary Figure 1.** Possible reason why Shaker dimers with W434F mutation (diamonds) in only one of their PD (all VSD^wt^) show different inactivation voltage dependences and kinetics depending on which protomer is mutated in its PD. Shaker dimers are represented in panels **a** and **b** with the protomers color coded in blue for protomer 1 and red for protomer 2. **a** shows Shaker dimer with the protomer 1 containing mutation PD^W434F^ and **b** shows dimers with that mutation in protomer 2. Note that since protomer 1 has a C-terminal that is not free, mutations in its PD may introduce slightly different features to the inactivation process as compared with the inactivation in dimers with the mutation in protomer 2 which has a free C-terminal.


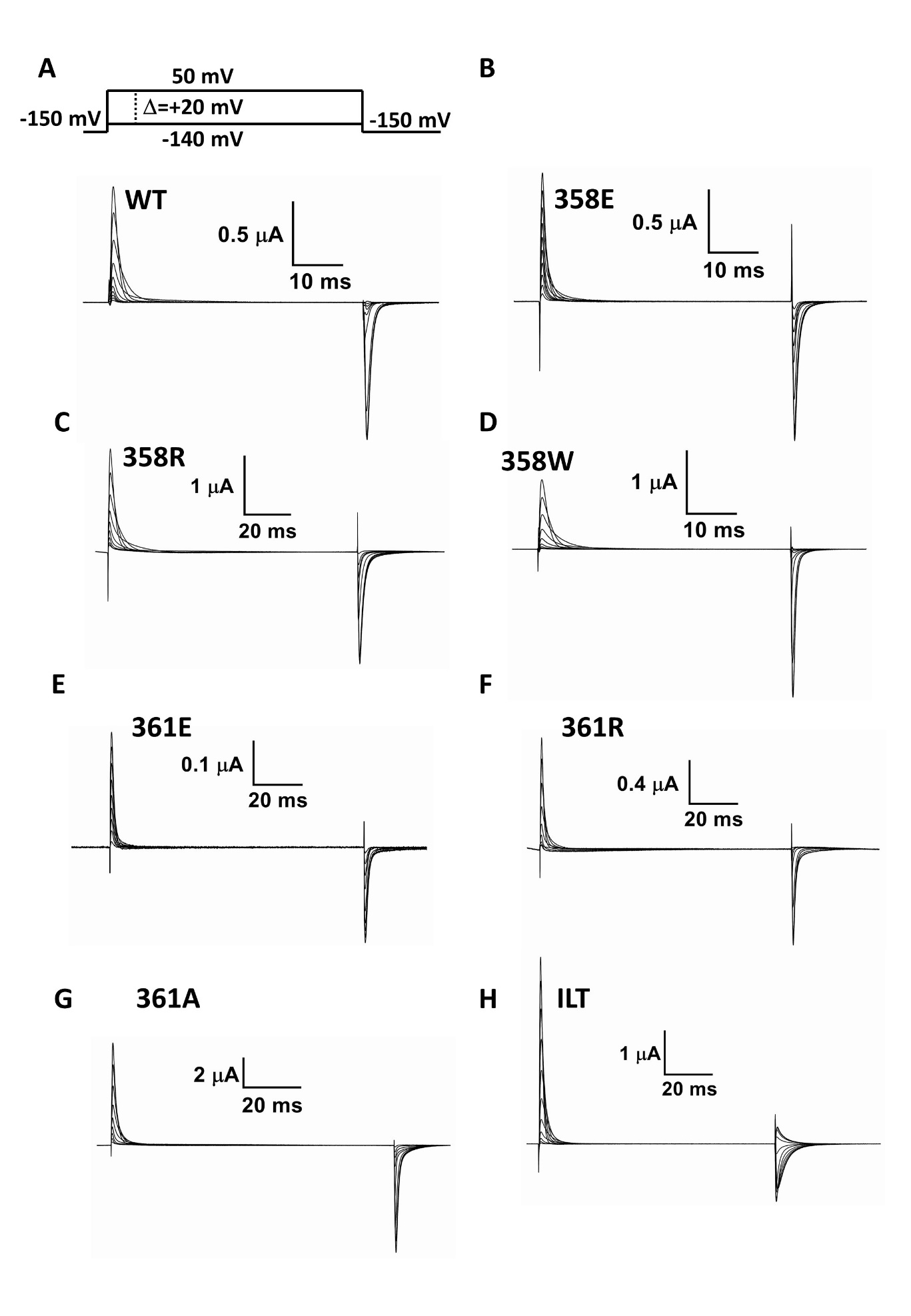


**Supplementary Figure 2.** **Gating currents in non-conductive Shaker dimers.** **A**, top panel, voltage protocol for gating currents recording; bottom panel, typical gating currents from non-conductive Shaker dimers bearing only wild-type VSD. **B-H**, gating currents from non-conductive Shaker dimers containing the indicated different mutations (**B**: 358E, **C**: 358R, **D**: 358W, **E**: 361E, **F**: 361R, **G**: 361A, **H**: ILT) in the VSD of protomer 2. These currents were recorded in response to the same voltage protocol shown in **A**, except for the recordings shown in **H** where the returning (final) voltage was -80 mV. Leak currents from all traces were subtracted offline. Bars indicating amplitude and time base are shown separately for each panel.


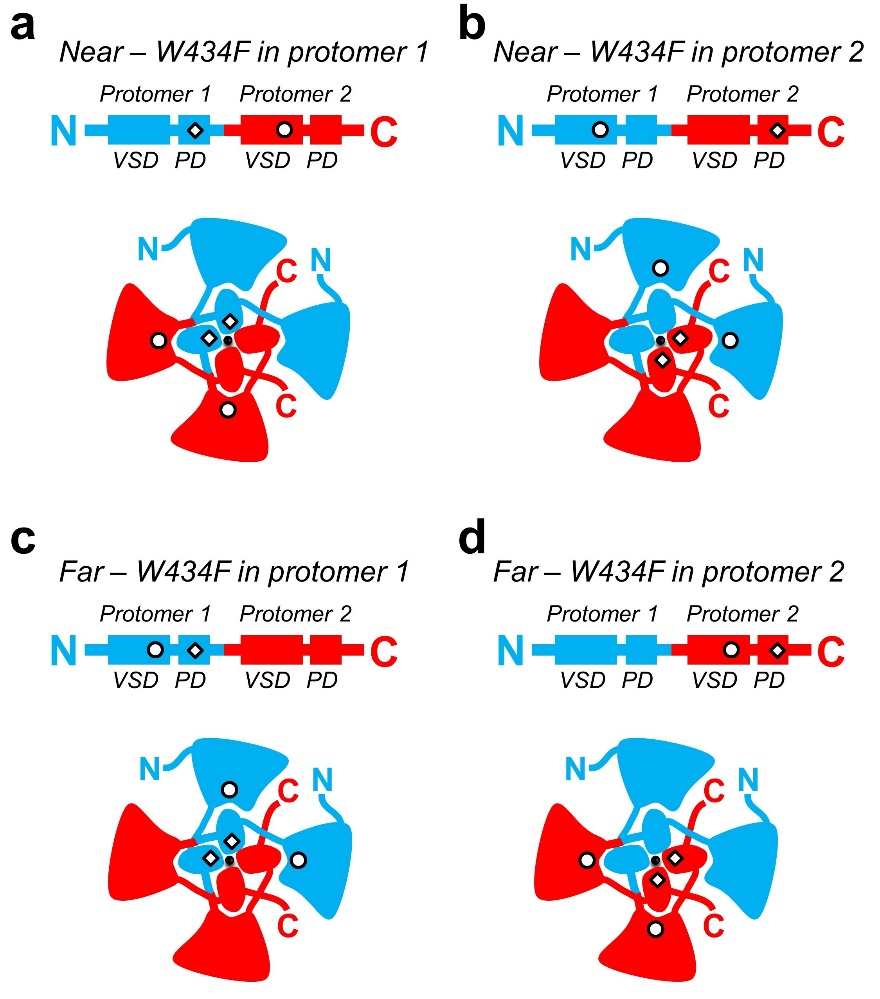


**Supplementary Figure 3.** **Speculative diagonally arranged dimerized Shaker proteins.** **a** and **b** show how the channels would look from the extracellular side if the mutations in VSD and PD are in different protomers as for near channels configuration. **c** and **d** show top views of channels putatively formed by proteins that were mutated in their VSD and PD in the same protomer as for far channels configuration. Note that the four channels shown in **a-d** are very similar in their VSD//PD interfaces, with the same four different interfaces in all of them.

**SUPPLEMENTARY NOTES**

**Supplementary Note 1**

The depletion experiments are done by using 0 K^+^ in both sides and 0 K^+^ in the microelectrode and by constantly pulsing so that there is outward current for each pulse. In fact, it is not possible to eliminate the internal K^+^ by just holding the membrane depolarized because slow inactivation prevents the outward flux of K^+^, even when internal perfusion is used. In the case of the 2X W434F, each pulse produces only a fast transient current, therefore it is very hard to deplete the internal K^+^ in a reasonable time before rundown, and this rundown is much faster in the perfused cut-open oocyte.

**Supplementary Note 2**

**Shaker cDNA (delta 6-46) sequence in pBSTa**

In the following cDNA sequence of pBSTa containing Shaker coding sequence, start codon and stop codon for Shaker translation are highlighted in black.

5’GGGAAATTGTAAACGTTAATATTTTGTTAAAATTCGCGTTAAATTTTTGTTAAATCAGCTCATTTTTTAACCAATAGGCCGAAATCGGCAAAATCCCTTATAAATCAAAAGAATAGACCGAGATAGGGTTGAGTGTTGTTCCAGTTTGGAACAAGAGTCCACTATTAAAGAACGTGGACTCCAACGTCAAAGGGCGAAAAACCGTCTATCAGGGCGATGGCCCACTACGTGAACCATCACCCTAATCAAGTTTTTTGGGGTCGAGGTGCCGTAAAGCACTAAATCGGAACCCTAAAGGGAGCCCCCGATTTAGAGCTTGACGGGGAAAGCCGGCGAACGTGGCGAGAAAGGAAGGGAAGAAAGCGAAAGGAGCGGGCGCTAGGGCGCTGGCAAGTGTAGCGGTCACGCTGCGCGTAACCACCACACCCGCCGCGCTTAATGCGCCGCTACAGGGCGCGTCGCGCCATTCGCCATTCAGGCTGCGCAACTGTTGGGAAGGGCGATGAATTCATCGGTGCGGGCCTCTTCGCTATTACGCCAGCTGGCGAAAGGGGGATGTGCTGCAAGGCGATTAAGTTGGGTAACGCCAGGGTTTTCCCAGTCACGACGTTGTAAAACGACGGCCAGTGAATTGTAATACGACTCACTATAGGGCGAATTGGGTACCGGGCCCCCCCTCGAGGTCGACGGTATCGATAAGCTTGCTTGTTCTTTTTGCAGAAGCTCAGAATAAACGCTCAACTTTGGCAGATCGCCACCATGGCCGCCGTTGCCCTGCGGGAGCAGCAGCTCCAGCGCAACTCCCTCGATGGTTACGGGTCTTTGCCCAAATTGAGCAGTCAAGACGAAGAAGGGGGGGCTGGTCATGGCTTTGGTGGCGGACCGCAACACTTTGAACCCATTCCTCACGATCATGATTTCTGCGAAAGAGTCGTTATAAATGTAAGCGGATTAAGGTTTGAGACACAACTACGTACGTTAAATCAATTCCCGGACACGCTGCTTGGGGATCCAGCTCGGAGATTACGGTACTTTGACCCGCTTAGAAATGAATATTTTTTTGACCGTAGTCGACCGAGCTTCGATGCGATTTTATACTATTATCAGAGTGGTGGCCGACTACGGAGACCGGTCAATGTCCCTTTAGACGTATTTAGTGAAGAAATAAAATTTTATGAATTAGGTGATCAAGCAATTAATAAATTCAGAGAGGATGAAGGCTTTATTAAAGAGGAAGAAAGACCATTACCGGATAATGAGAAACAGAGAAAAGTCTGGCTGCTCTTCGAGTATCCAGAAAGTTCGCAAGCCGCCAGAGTTGTAGCCATAATTAGTGTATTTGTTATATTGCTATCAATTGTTATATTTTGTCTAGAAACATTACCCGAATTTAAGCATTACAAGGTGTTCAATACAACAACAAATGGCACAAAAATCGAGGAAGACGAGGTGCCTGACATCACAGATCCTTTCTTCCTTATAGAAACGTTATGCATTATTTGGTTTACATTTGAACTAACTGTCAGGTTCCTCGCATGTCCGAACAAATTAAATTTCTGCAGGGATGTCATGAATGTTATCGACATAATCGCCATCATTCCGTACTTTATAACACTAGCGACTGTCGTTGCCGAAGAGGAGGATACGTTAAATCTTCCAAAAGCGCCAGTCAGTCCACAGGACAAGTCATCGAATCAGGCTATGTCCTTGGCAATATTACGAGTGATACGATTAGTTCGAGTATTTCGAATATTTAAGTTATCTAGGCATTCGAAGGGTTTACAGATCTTAGGACGAACTCTGAAAGCCTCAATGCGGGAATTAGGTTTACTTATATTTTTCTTATTTATAGGCGTCGTACTCTTCTCATCGGCGGTTTATTTTGCGGAAGCTGGAAGCGAAAATTCCTTCTTCAAGTCCATACCCGATGCATTTTTCTGGGCGGTGGTTACCATGACCACCGTTGGATATGGTGACATGACACCCGTCGGCGTTTGGGGCAAGATTGTGGGATCACTTTGTGCCATTGCTGGCGTGCTGACCATCGCACTGCCGGTGCCGGTCATCGTCAGCAATTTCAACTACTTCTATCACCGCGAAACGGATCAGGAGGAGATGCAGAGCCAGAACTTTAATCACGTTACTAGTTGTCCATATTTGCCCGGGACATTAGTAGGTCAACACATGAAGAAATCATCATTGTCTGAGTCCTCATCGGATATGATGGATTTGGACGATGGTGTCGAGTCCACGCCGGGATTGACAGAAACACATCCTGGACGCAGTGCGGTGGCTCCATTTTTGGGAGCCCAGCAGCAGCAGCAACAACCGGTAGCATCCTCACTGTCGATGTCGATCGACAAACAACTGCAGCACCCACTGCAGCAGCTGACGCAGACGCAACTGTACCAACAGCAGCAACAGCAGCAGCAGCAGCAGCAAAACGGCTTCAAGCAGCAGCAGCAACAGACGCAGCAGCAGCTGCAACAGCAACAGTCCCACACAATAAACGCAAGTGCAGCAGCGGCGACGAGCGGCAGCGGCAGTAGCGGTCTCACCATGAGGCACAATAATGCCCTGGCCGTTAGTATCGAGACCGACGTTTGACTACTGGTGGCAGATCTGGTTACGTTACCACTAAACCAGCCTCAAGAACACCCGAATGGAGTCTCTAAGCTACATAATACCAACTTACACTTTACAAAATGTTGTCCCCCAAAATGTAGCCATTCGTATCTGCTCCTAATAAAAAGAAAGTTTCTTCACATTCTAAAAAAAAAAAAAAAAAAAAAAAAAAAAAAAAACCCCCCCCCCCCCCCCCCTGCAGCCCCTAGAGC’GGCCGCCACCGCGGTGGAGCTCCAGCTTTTGTTCCCTTTAGTGAGGGTTAATTCCGAGCTTGGCGTAATCATGGTCATAGCTGTTTCCTGTGTGAAATTGTTATCCGCTCACAATTCCACACAACATACGAGCCGGAAGCATAAAGTGTAAAGCCTGGGGTGCCTAATGAGTGAGCTAACTCACATTAATTGCGTTGCGCTCACTGCCCGCTTTCCAGTCGGGAAACCTGTCGTGCCAGCTGCATTAATGAATCGGCCAACGCGCGGGGAGAGGCGGTTTGCGTATTGGGCGCTCTTCCGCTTCCTCGCTCACTGACTCGCTGCGCTCGGTCGTTCGGCTGCGGCGAGCGGTATCAGCTCACTCAAAGGCGGTAATACGGTTATCCACAGAATCAGGGGATAACGCAGGAAAGAACATGTGAGCAAAAGGCCAGCAAAAGGCCAGGAACCGTAAAAAGGCCGCGTTGCTGGCGTTTTTCCATAGGCTCCGCCCCCCTGACGAGCATCACAAAAATCGACGCTCAAGTCAGAGGTGGCGAAACCCGACAGGACTATAAAGATACCAGGCGTTTCCCCCTGGAAGCTCCCTCGTGCGCTCTCCTGTTCCGACCCTGCCGCTTACCGGATACCTGTCCGCCTTTCTCCCTTCGGGAAGCGTGGCGCTTTCTCATAGCTCACGCTGTAGGTATCTCAGTTCGGTGTAGGTCGTTCGCTCCAAGCTGGGCTGTGTGCACGAACCCCCCGTTCAGCCCGACCGCTGCGCCTTATCCGGTAACTATCGTCTTGAGTCCAACCCGGTAAGACACGACTTATCGCCACTGGCAGCAGCCACTGGTAACAGGATTAGCAGAGCGAGGTATGTAGGCGGTGCTACAGAGTTCTTGAAGTGGTGGCCTAACTACGGCTACACTAGAAGGACAGTATTTGGTATCTGCGCTCTGCTGAAGCCAGTTACCTTCGGAAAAAGAGTTGGTAGCTCTTGATCCGGCAAACAAACCACCGCTGGTAGCGGTGGTTTTTTTGTTTGCAAGCAGCAGATTACGCGCAGAAAAAAAGGATCTCAAGAAGATCCTTTGATCTTTTCTACGGGGTCTGACGCTCAGTGGAACGAAAACTCACGTTAAGGGATTTTGGTCATGAGATTATCAAAAAGGATCTTCACCTAGATCCTTTTAAATTAAAAATGAAGTTTTAAATCAATCTAAAGTATATATGAGTAAACTTGGTCTGACAGTTACCAATGCTTAATCAGTGAGGCACCTATCTCAGCGATCTGTCTATTTCGTTCATCCATAGTTGCCTGACTCCCCGTCGTGTAGATAACTACGATACGGGAGGGCTTACCATCTGGCCCCAGTGCTGCAATGATACCGCGAGACCCACGCTCACCGGCTCCAGATTTATCAGCAATAAACCAGCCAGCCGGAAGGGCCGAGCGCAGAAGTGGTCCTGCAACTTTATCCGCCTCCATCCAGTCTATTAATTGTTGCCGGGAAGCTAGAGTAAGTAGTTCGCCAGTTAATAGTTTGCGCAACGTTGTTGCCATTGCTACAGGCATCGTGGTGTCACGCTCGTCGTTTGGTATGGCTTCATTCAGCTCCGGTTCCCAACGATCAAGGCGAGTTACATGATCCCCCATGTTGTGCAAAAAAGCGGTTAGCTCCTTCGGTCCTCCGATCGTTGTCAGAAGTAAGTTGGCCGCAGTGTTATCACTCATGGTTATGGCAGCACTGCATAATTCTCTTACTGTCATGCCATCCGTAAGATGCTTTTCTGTGACTGGTGAGTACTCAACCAAGTCATTCTGAGAATAGTGTATGCGGCGACCGAGTTGCTCTTGCCCGGCGTCAATACGGGATAATACCGCGCCACATAGCAGAACTTTAAAAGTGCTCATCATTGGAAAACGTTCTTCGGGGCGAAAACTCTCAAGGATCTTACCGCTGTTGAGATCCAGTTCGATGTAACCCACTCGTGCACCCAACTGATCTTCAGCATCTTTTACTTTCACCAGCGTTTCTGGGTGAGCAAAAACAGGAAGGCAAAATGCCGCAAAAAAGGGAATAAGGGCGACACGGAAATGTTGAATACTCATACTCTTCCTTTTTCAATATTATTGAAGCATTTATCAGGGTTATTGTCTCATGAGCGGATACATATTTGAATGTATTTAGAAAAATAAACAAATAGGGGTTCCGCGCACATTTCCCCGAAAAGTGCCACCT 3’

**Supplementary Note 3**

**Primers used for mutations**

W434F mutation

FWD 5’ CCGATGCATTTTTCTGGGCGGTGGTTAC 3’

REV 5’ GTAACCACCGCCCAGAAAAATGCATCGG 3’

ILT triple mutation

FWD 5’ GATTAGTTCGAATCTTTCGACTGTTTAAGTTAACCAGGCATTCGAAG 3’

REV 5’ CTTCGAATGCCTGGTTAACTTAAACAGTCGAAAGATTCGAACTAATC 3’

358E mutation

FWD 5’ GAATCAGGCTATGTCCGAGGCAATATTACGAGTG 3’

REV 5’ CACTCGTAATATTGCCTCGGACATAGCCTGATTC 3’

358R mutation

FWD 5’ GAATCAGGCTATGTCCCGAGCAATATTACGAGTG 3’

REV 5’ CACTCGTAATATTGCTCGGGACATAGCCTGATTC 3’

358W mutation

FWD 5’ CAGGCTATGTCCTGGGCAATATTACG 3’

REV 5’ CGTAATATTGCCCAGGACATAGCCTG 3’

361E mutation

FWD 5’ CCTTGGCAATAGAGCGAGTGATACG 3’

REV 5’ CGTATCACTCGCTCTATTGCCAAGG 3’

361R mutation

FWD 5’ CCTTGGCAATACGACGAGTGATACG 3’

REV 5’ CGTATCACTCGTCGTATTGCCAAGG 3’

361A mutation

FWD 5’ CCTTGGCAATAGCTCGAGTGATACG 3’

REV 5’ CGTATCACTCGAGCTATTGCCAAGG 3’

412V mutation

FWD 5’ CGTACTCTTCTCAGTTGCGGTTTATTTTG 3’

REV 5’ CAAAATAAACCGCAACTGAGAAGAGTACG 3’

**Supplementary Note 4**

**Primers used for dimerization**

NcoI to AvrII+G swap

FWD 5’ GCAGATCGCCACCTAGGGCCGCCGTTGCCC 3’

REV 5’ GGGCAACGGCGGCCCTAGGTGGCGATCTGC 3’

Stop to AvrII swap

FWD 5’ CGAGACCGACGTTCCTAGGCTACTGGTGGCAG 3’

REV 5’ CTGCCACCAGTAGCCTAGGAACGTCGGTCTCG 3’
