# Supplementary material for: Noncanonical mechanism of voltage sensor coupling to pore revealed by tandem dimers of Shaker
Source: Nat Commun. 2019 Aug 8;10:3584. doi: 10.1038/s41467-019-11545-7 (PMC6687735; doi:10.1038/s41467-019-11545-7)
Supplement: Supplementary file 2 — Reporting Summary [file 41467_2019_11545_MOESM2_ESM.pdf]

nature research

Corresponding author(s): NCOMMS-18-24387B

Last updated by author(s): 7-8-19

Reporting Summary

Nature Research wishes to improve the reproducibility of the work that we publish. This form provides structure for consistency and transparency in reporting. For further information on Nature Research policies, see Authors & Referees and the Editorial Policy Checklist.

Statistics

For all statistical analyses, confirm that the following items are present in the figure legend, table legend, main text, or Methods section.

n/a	Confirmed	
☐	☒	The exact sample size (*n*) for each experimental group/condition, given as a discrete number and unit of measurement
☐	☒	A statement on whether measurements were taken from distinct samples or whether the same sample was measured repeatedly
☒	☐	The statistical test(s) used AND whether they are one- or two-sided<br>*Only common tests should be described solely by name; describe more complex techniques in the Methods section.*
☒	☐	A description of all covariates tested
☒	☐	A description of any assumptions or corrections, such as tests of normality and adjustment for multiple comparisons
☐	☒	A full description of the statistical parameters including central tendency (e.g. means) or other basic estimates (e.g. regression coefficient) AND variation (e.g. standard deviation) or associated estimates of uncertainty (e.g. confidence intervals)
☒	☐	For null hypothesis testing, the test statistic (e.g. *F*, *t*, *r*) with confidence intervals, effect sizes, degrees of freedom and *P* value noted<br>*Give P values as exact values whenever suitable.*
☒	☐	For Bayesian analysis, information on the choice of priors and Markov chain Monte Carlo settings
☒	☐	For hierarchical and complex designs, identification of the appropriate level for tests and full reporting of outcomes
☒	☐	Estimates of effect sizes (e.g. Cohen's *d*, Pearson's *r*), indicating how they were calculated

Our web collection on statistics for biologists contains articles on many of the points above.

Software and code

Policy information about availability of computer code

Data collection	In-house software was used to collect the electrophysiologycal data (ionic or gating currents) from the experiments for this work.
Data analysis	In-house software was used to analyze the data. Microsoft Excel was used to organize the data and for the calculation of means and standard error of the means. GraphPad Prism (GraphPad Software, Inc.) was used to plot the data.

For manuscripts utilizing custom algorithms or software that are central to the research but not yet described in published literature, software must be made available to editors/reviewers. We strongly encourage code deposition in a community repository (e.g. GitHub). See the Nature Research guidelines for submitting code & software for further information.

Data

Policy information about availability of data

All manuscripts must include a data availability statement. This statement should provide the following information, where applicable:

- Accession codes, unique identifiers, or web links for publicly available datasets
- A list of figures that have associated raw data
- A description of any restrictions on data availability

The data that support the findings of this study are available on reasonable request from the corresponding author FB.

Field-specific reporting

Please select the one below that is the best fit for your research. If you are not sure, read the appropriate sections before making your selection.

☒ Life sciences ☐ Behavioural & social sciences ☐ Ecological, evolutionary & environmental sciences

For a reference copy of the document with all sections, see nature.com/documents/nr-reporting-summary-flat.pdf

Life sciences study design

All studies must disclose on these points even when the disclosure is negative.

Sample size	We determined the sample size to be between 4-6 repetitions of the same experiment, independently. No repeated measurement was performed in the same oocyte.
Data exclusions	No data was excluded from analysis other than those that clearly indicated unsuccessful expression of the protein by the oocytes. We defined those cases when the amplitude of the current peak to be recorded was smaller than 500 nA.
Replication	Different batches of oocytes were used for the heterologous expression of the channels.
Randomization	The frogs we used as oocytes donors were wild type frogs and they were selected from our colony for the day of oocytes surgical extraction without any criteria that would bias the outcomes. We believe this introduces a high level of randomization to our assays.
Blinding	We did not performed blind experiments.

Reporting for specific materials, systems and methods

We require information from authors about some types of materials, experimental systems and methods used in many studies. Here, indicate whether each material, system or method listed is relevant to your study. If you are not sure if a list item applies to your research, read the appropriate section before selecting a response.

Materials & experimental systems

n/a	Involved in the study	
☒	☐	Antibodies
☒	☐	Eukaryotic cell lines
☒	☐	Palaeontology
☐	☒	Animals and other organisms
☒	☐	Human research participants
☒	☐	Clinical data

Methods

n/a	Involved in the study	
☒	☐	ChIP-seq
☒	☐	Flow cytometry
☒	☐	MRI-based neuroimaging

Animals and other organisms

Policy information about studies involving animals; ARRIVE guidelines recommended for reporting animal research

Laboratory animals	We used Xenopus laevis females as oocytes donors.
Wild animals	No wild animal was used in the present study.
Field-collected samples	No field-collected sample was used in the present study.
Ethics oversight	The University of Chicago IACUC approved the protocols followed by the present study.

Note that full information on the approval of the study protocol must also be provided in the manuscript.
